# Chinese herbal medicine Du-Huo-Ji-Sheng-decoction for knee osteoarthritis: A protocol for systematic review and meta-analysis

**DOI:** 10.1097/MD.0000000000024413

**Published:** 2021-01-22

**Authors:** Ji-hui Cao, Da-gang Feng, Yan-zhi Wang, Hai-yan Zhang, Yu-dong Zhao, Zai-hui Sun, Shu-gui Feng, Yi Chen, Ming-shuang Zhu

**Affiliations:** a School of Clinical Medicine, Chengdu University of Traditional Chinese Medicine; b Affiliated Hospital of Chengdu University of Traditional Chinese Medicine, Sichuan, China.

**Keywords:** Du-Huo-Ji-Sheng-decoction, knee osteoarthritis, protocol, systematic review

## Abstract

**Background:**

As a classic prescription for treating knee osteoarthritis, Du-Huo-Ji-Sheng-decoction has been widely recognized for its clinical efficacy. The purpose of this systematic review and meta-analysis is to evaluate the effectiveness and safety of Du-Huo-Ji-Sheng-decoction in the treatment of knee osteoarthritis.

**Methods:**

The following databases will be searched from January 2011 to December 2020: PubMed, Embase, Web of Science, Cochrane Library, Chinese Biomedical Medical Database, China National Knowledge Infrastructure, Chinese Science and Technology Periodical Database, and Wanfang Database. Statistical analysis will be processed by RevMan V.5.3 software.

**Results:**

This study will provide an assessment of the current state of DHJSD in the treatment of KOA, aiming to show the efficacy and safety of DHJSD.

**Conclusion:**

This study will provide evidence to judge whether DHJSD is an effective intervention for KOA.

## 1 Introduction

(KOA) is a common disease in orthopedics; it has the characteristics of high prevalence, wide range of lesions, and severe late dysfunction.^[1–3]^ Among people over 60 years old, the incidence of the disease is about 10% in men and 18% in women. The knee joint is the main body joint with the highest incidence of osteoarthritis.^[4,5]^ The main clinical manifestations of KOA are knee joint pain limited mobility, and a high disability rate, which has a serious impact on the quality of life of patients.^[6,7]^ With the aging of the global population, the incidence of the disease is increasing year by year. The trend has brought a huge economic burden to the society.^[8–10]^ The basic principles of clinical treatment are to relieve pain, delay disease progression, and improve knee joint function.^[11]^ At present, the disease cannot be cured, and there is no specific treatment drug, so the treatment methods to delay the disease are particularly important.^[12,13]^ Traditional Chinese medicine has unique advantages in the treatment of KOA. Among them, the importance and advantages of (DHJSD) in the treatment of KOA are well recognized by ancient and modern doctors.^[14,15]^ Although it has been widely used in the treatment of KOA in clinical practice, there is no systematic review to explore the efficacy of DHJSD on KOA. Therefore, the purpose of this study is to evaluate the efficacy and safety of DHJSD on KOA.

## 2 Methods

This study has been registered on on INPLASY. The registration DOI of this protocol is 2020120095. We will follow the Preferred Reporting Item for Systematic Review and Meta-Analysis to perform this study.

### 2.1 Inclusion criteria for study selection

#### 2.1.1 Types of studies

All randomized controlled trial (RCT) of DHJSD in the treatment of KOA will be included language is limited to English and Chinese. Observational studies and non-RCTs will be excluded. There will be no restriction on publication date.

#### 2.1.2 Types of participants

This review will consider all relevant studies DHJSD in the treatment of KOA. There are no restrictions of gender, race, or age.

#### 2.1.3 Types of interventions

##### 2.1.3.1 Experimental interventions

The intervention method of the experimental group is DHJSD. There are no restrictions on administration time, frequency, and dosage form. Research combined with other interventions (such as acupuncture, massage) will be excluded.

##### 2.1.3.2 Control interventions

Treatments other than DHJSD (eg, gui zhi ge gen Decoction, placebo, rehabilitation treatments). There are no restrictions on administration time, frequency, and dosage form.

#### 2.1.4 Outcome measurements

The main results will be Hospital for Special Surgery score.^[16]^ Secondary results will be visual analogue scale scores and Western Ontario and McMaster Universities Osteoarthritis Index.^[17,18]^

#### 2.1.5 Exclusion criteria

Nonrandomized controlled trials, quasi randomized controlled trials, animal studies, crossover trials, conference articles, case reports, expert experience, research combined with other interventions (such as acupuncture, massage) will be excluded.

### 2.2 Search methods for the identification of studies

The following databases will be searched: PubMed, Embase, Web of Science, Cochrane Library, Chinese Biomedical Medical Database, China National Knowledge Infrastructure, Chinese Science and Technology Periodical Database, and Wanfang Database. We will search the records in these databases covering January 2011 to December 2020. Search terms consist of disease (knee osteoarthritis or knee osteoarthritides or knee joint osteoarthritis or knee pain or osteoarthritis of knee or knee arthritis) and intervention (Du-huo-ji-sheng-decoction or Du-huo-ji-sheng-Tang) and research types (randomized controlled trial). Specific search strategy of PubMed is shown in Table 1.

**Table 1:** Search strategy for PubMed database.

No	Search items
#1	Knee osteoarthritis. ti, ab
#2	Knee osteoarthritides. ti, ab
#3	Knee pain. ti, ab
#4	Knee joint osteoarthritis. ti, ab
#5	Knee arthritis. ti, ab
#6	Osteoarthritis of knee. ti, ab
#7	#1 or #2–6
#8	Duhuojisheng decoction. ti, ab
#9	Duhuojisheng Tang. ti, ab
#10	#8 or #9
#11	Randomized controlled trial. ti, ab
#12	Random trials. ti, ab
#13	Controlled clinical trial. ti, ab
#14	#11or #12 #13
#15	#7and #10 and #14

### 2.3 Data collection and analysis

#### 2.3.1 Study identification

**Figure 1:**
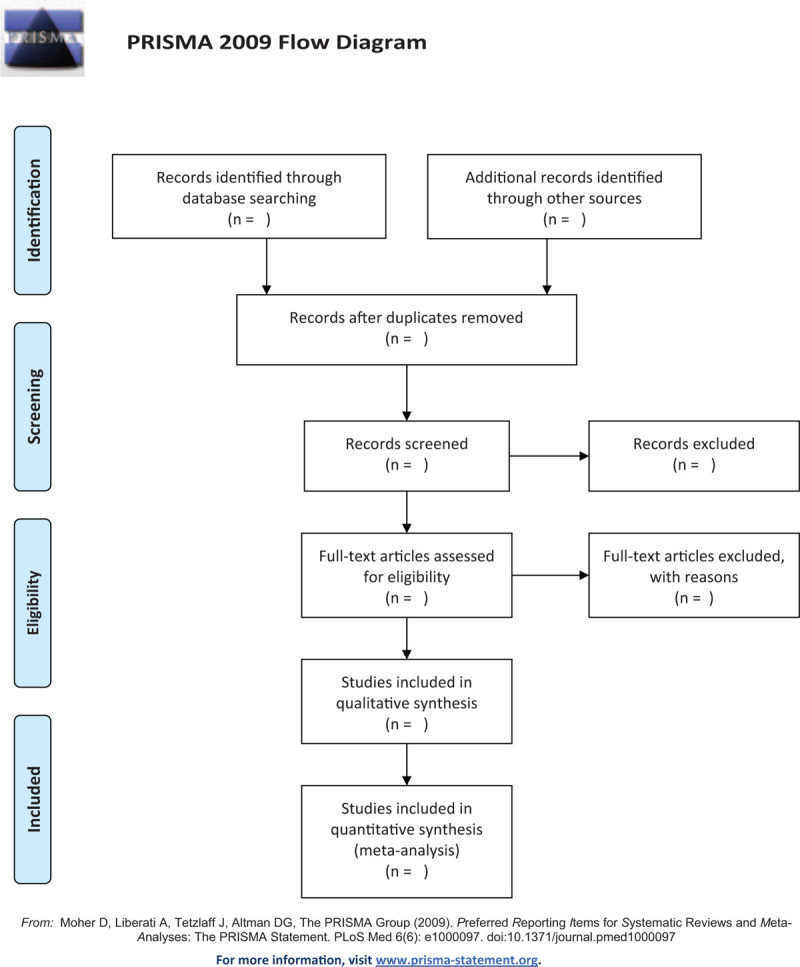
Preferred Reporting Item for Systematic Review and Meta-Analysis flowchart.

We will use EndNote (X9;Clarivate Analytics, Philadelphia) software to manage the records of searched electronic databases. The initial selection will involve scanning of the titles and abstracts of the retrieved studies. The full text of relevant studies will then be reviewed in accordance with the inclusion criteria by 2 authors (DGF and YZW). Potentially relevant articles will be reviewed independently by 2 authors to determine if they meet the criteria. Any disagreement between authors will be resolved by consensus with the third author (HYZ). The study selection procedure will follow and be recorded in the Preferred Reporting Items for Systematic Reviews and Meta-analysis flow chart (see Fig. 1).

#### 2.3.2 Data extraction

Data will be extracted from the included studies using a standardized data extraction tool by 2 independent reviewers. The extracted information will mainly include study design, the first author, year of publication, participant characteristics, sample size, type of intervention (e.g., dosage, regimen, administration method, and herbal composition of prescription), results, and adverse events. Extracted data will be presented as included study summary table.

### 2.4 Risk of bias assessment

The risk of bias in included studies will be assessed independently by 2 reviewers (YDZ and ZHS) using the Cochrane Risk of Bias Tool, with any disagreement resolved by consensus or by discussion with a third reviewer (MSZ). The tool has 7 domains, which includes random sequence generation, blinding of outcome assessment, blinding of participants and personnel, allocation concealment, selective reporting, incomplete outcome data, and other sources of bias. The risk of bias in each item is rated as “high”, “low risk”, or “unclear of bias”.^[19]^

### 2.5 Dealing with missing data

When data are missing, we will look for the reason. Then, we will contact the corresponding author to obtain and verify the data if possible. If this does not work, we will only analyze the available data.

### 2.6 Data analysis

In order to summarize the therapeutic effect of DHJSD on KOA, we will use Review Manager Version 5.3 (RevMan V5.3, Cochrane Center, Copenhagen, Denmark) to evaluate the difference between the intervention group and the control group in the study. The treatment effect will be assessed by the mean value. The difference of 95% confidence interval will be measured. In processing dichotomy data, we will consider the processing effect as the relative risk of 95% confidence interval; other binary data will be converted to RR.

Then the χ^2^ test will be used to determine whether there is heterogeneity between the studies (the test level is α = 0.1). If *P* > .1 or I^2^ ≤ 50%, it is considered that there is no heterogeneity among multiple studies, and a fixed-effect model can be used to conduct a meta-analysis of the combined effect size. If *P* ≤ .1 or I^2^ > 50%, it is considered that there is obvious heterogeneity between multiple studies, and random effects models can be used to combine effect size and analysis. If the heterogeneity is too large (I^2^ ≥ 75%), it will not be combined and only a descriptive analysis will be performed.

### 2.7 Assessment of reporting biases

If more than 10 RCTs are included in this study, we will use a funnel chart to assess publication bias. Otherwise, The Egger test will be used by us for evaluation.^[20]^

### 2.8 Subgroup analysis

If the number of included studies is sufficient, we will analyze the subgroups according to the age, gender, course of treatment, intervention methods, and dosage forms of the control group.

### 2.9 Sensitivity analysis

In order to verify the stability of the main results, we will conduct a sensitivity analysis based on the study design, sample size, methodological quality, and the impact of missing data included in the study.

### 2.10 Grading the quality of evidence

We will assess the quality of evidence by the Grading of Recommendations Assessment, Development, and Evaluation and rate it into 4 levels (high, medium, low, or very low).^[21]^

### 2.11 Ethics and dissemination

In the review of this system, we will collect data from published studies. Based on this, the study does not require ethical approval.

## 3 Discussion

The pathogenesis of KOA is quite complex. It is generally believed to be closely related to factors such as genetics, obesity, overwork, and rest.^[22–26]^ Pathogenic factors often involve cartilage degenerative changes and uneven joint force, which causes pain in patients.^[27,28]^ The inflammatory damage of the cartilage layer in the superficial area is inseparable from the increase of age, and it will form osteoarthritis changes in the long run.^[29]^ Although a large number of studies have reported the effect of DHJSD in the treatment of KOA, there is still a lack of comprehensive and systematic reviews and research evidence on the effectiveness and safety of DHJSD.^[15,30]^ Therefore, this study aims to explore the effectiveness and safety of DHJSD for KOA patients. The results of this study will provide a more reliable basis for DHJSD to relieve the pain of patients and improve the quality of life, so as to better utilize the advantages and characteristics of traditional Chinese medicine, provide modern clinical services, and provide evidence-based medicine recommendations for DHJSD in the treatment of KOA.

## Author contributions

**Conceptualization:** Ming-shuang Zhu.

**Data curation:** Ji-hui Cao, Da-gang Feng, Yan-zhi Wang.

**Methodology:** Ming-shuang Zhu.

**Resources:** Hai-yan Zhang, Yu-dong Zhao, Zai-hui Sun.

**Supervision:** Shu-gui Feng, Yi Chen.

**Writing – original draft:** Ji-hui Cao.

**Writing – review & editing:** Ming-shuang Zhu.
